# Sequence artefacts in a prospective series of formalin-fixed tumours tested for mutations in hotspot regions by massively parallel sequencing

**DOI:** 10.1186/1755-8794-7-23

**Published:** 2014-05-13

**Authors:** Stephen Q Wong, Jason Li, Angela Y-C Tan, Ravikiran Vedururu, Jia-Min B Pang, Hongdo Do, Jason Ellul, Ken Doig, Anthony Bell, Grant A MacArthur, Stephen B Fox, David M Thomas, Andrew Fellowes, John P Parisot, Alexander Dobrovic

**Affiliations:** 1 Department of Pathology, Peter MacCallum Cancer Centre, East Melbourne, Victoria 3002, Australia; 2 Bioinformatics, Cancer Research Division, Peter MacCallum Cancer Centre, East Melbourne, Victoria 3002, Australia; 3 Department of Pathology, The University of Melbourne, Parkville, Victoria 3010, Australia; 4 Translational Genomics and Epigenomics Laboratory, Ludwig Institute for Cancer Research, The Olivia Newton-John Cancer and Wellness Centre, Heidelberg, Victoria 3084, Australia; 5 Sir Peter MacCallum Department of Oncology, The University of Melbourne, Parkville, Victoria 3010, Australia; 6 Division of Cancer Research, Peter MacCallum Cancer Centre, St Andrews Place, East Melbourne, Victoria 3002, Australia; 7 The Kinghorn Cancer Centre and Garvan Institute, Victoria Street, Darlinghurst 2010, New South Wales, Australia

## Abstract

**Background:**

Clinical specimens undergoing diagnostic molecular pathology testing are fixed in formalin due to the necessity for detailed morphological assessment. However, formalin fixation can cause major issues with molecular testing, as it causes DNA damage such as fragmentation and non-reproducible sequencing artefacts after PCR amplification. In the context of massively parallel sequencing (MPS), distinguishing true low frequency variants from sequencing artefacts remains challenging. The prevalence of formalin-induced DNA damage and its impact on molecular testing and clinical genomics remains poorly understood.

**Methods:**

The Cancer 2015 study is a population-based cancer cohort used to assess the feasibility of mutational screening using MPS in cancer patients from Victoria, Australia. While blocks were formalin-fixed and paraffin-embedded in different anatomical pathology laboratories, they were centrally extracted for DNA utilising the same protocol, and run through the same MPS platform (Illumina TruSeq Amplicon Cancer Panel). The sequencing artefacts in the 1-10% and the 10-25% allele frequency ranges were assessed in 488 formalin-fixed tumours from the pilot phase of the Cancer 2015 cohort. All blocks were less than 2.5 years of age (mean 93 days).

**Results:**

Consistent with the signature of DNA damage due to formalin fixation, many formalin-fixed samples displayed disproportionate levels of C>T/G>A changes in the 1-10% allele frequency range. Artefacts were less apparent in the 10-25% allele frequency range. Significantly, changes were inversely correlated with coverage indicating high levels of sequencing artefacts were associated with samples with low amounts of available amplifiable template due to fragmentation. The degree of fragmentation and sequencing artefacts differed between blocks sourced from different anatomical pathology laboratories. In a limited validation of potentially actionable low frequency mutations, a *NRAS* G12D mutation in a melanoma was shown to be a false positive.

**Conclusions:**

These findings indicate that DNA damage following formalin fixation remains a major challenge in laboratories working with MPS. Methodologies that assess, minimise or remove formalin-induced DNA damaged templates as part of MPS protocols will aid in the interpretation of genomic results leading to better patient outcomes.

## Background

Advances in genomic technologies are improving the capability to arrive at a more informed decision on how to treat a patient [1,2]. In particular, a large number of genes can be screened for actionable changes using massively parallel sequencing (MPS) approaches [3,4]. Accurate and reliable interpretation of this new type of genomic data is therefore critical in deciding the appropriate course of management for patients.

Most DNA for genetic testing from cancer patients is extracted from formalin-fixed paraffin-embedded tumour biopsies, where the primary intent is to preserve tumour cellular structure for histological examination and diagnosis [5]. However, the formalin fixation process is detrimental to downstream genomic applications, causing issues, such as DNA cross-linking to DNA and proteins, that can stall polymerases and DNA-DNA crosslinks that can inhibit denaturation [6].

A common form of DNA damage induced by formalin fixation is fragmentation, which can lead to low amounts of amplifiable template for PCR amplification. Fragmentation of DNA is caused by a number of factors during the fixation process, e.g. low pH formalin over time increases the rate of apurinic/apyrimidinic site formation and eventually decomposition and fragmentation [7]. Long-term storage of formalin-fixed blocks can also induce fragmentation due to exposure to environmental conditions [8-10].

Another prominent type of DNA damage that occurs commonly in formalin-fixed tissues is the hydrolytic deamination of cytosine to form uracil (or thymine if the cytosine is methylated). This results in non-reproducible C>T/G>A sequencing artefacts that are observed after PCR amplification when using formalin-fixed and paraffin-embedded (FFPE) DNA [11-13].

Recently, we assessed an amplicon-based MPS technology and showed that C>T and G>A changes were the most prominent sequence errors in three formalin-fixed lung squamous cell carcinoma samples [12]. While such artefacts occur in many formalin-fixed samples, we have found this to be more pronounced in highly fragmented samples. Due to stochastic effects, the low template numbers increase the probability of occurrence of template artefacts [14].

Many studies have reported the feasibility of using DNA from formalin-fixed material using both conventional PCR-based and MPS technologies [4,15,16]. Fragmentation can be a rate-limiting factor in amplicon-based approaches especially those that use longer amplicons. Shorter amplicons permit fragmented DNA from formalin-fixed material to be used more successfully.

Some studies have reported elevated numbers of sequence artefacts [12,16-19] whereas others reported little evidence of artefacts appearing in FFPE samples [15,20]. The small number of samples assessed as well as the varying age of biopsies, degree of fixation and sequencing technologies used makes it difficult to know how important sequence artefacts are as a source of error in relatively fresh FFPE samples.

This study assessed the prevalence of DNA fragmentation and sequencing artefacts from a large cohort of FFPE tumours using a uniform approach whereby all blocks were of similar age, were extracted in the same manner and were run through the same MPS platform. The originating anatomical pathology laboratory was also recorded to determine if variation in operating practices could affect sequencing artefacts and DNA fragmentation.

## Methods

### Patients and cell lines

Cancer 2015 is a large-scale, prospective, longitudinal, multi-site cohort study of incident cancers in the Victorian population. The aim of the Cancer 2015 study is to classify cancers molecularly using MPS to promote more targeted treatment of cancer patients and improve patient survival and outcomes. An initial pilot phase was established to determine the feasibility of adopting MPS for the diagnostic mutational profiling of tumours. This study was approved by the Human Research Ethics Committees at the Peter MacCallum Cancer Centre, Royal Melbourne, Cabrini, Geelong and Warrnambool Hospitals, all located within the state of Victoria, Australia. All patients provided informed consent to participate in this study. Formalin-fixed, paraffin-embedded (FFPE) tumour blocks or unstained sections from FFPE tumour blocks were acquired from anatomical pathology laboratories performing the diagnosis and sent to the Peter MacCallum Cancer Centre Pathology department. Epidemiological and clinical variables were collected from each patient. As controls representing good quality DNA, the cell lines NCI-H1975 (H1975) and HL-60 were also sequenced.

### DNA extraction

All blocks/sections were received at the Peter MacCallum Cancer Centre Pathology department for DNA extraction. The age of the blocks/sections was determined by identifying the duration between the date of fixation (date of surgery) and the date of extraction (arrival date at the Peter MacCallum Cancer Centre). Up to ten unstained tumour sections of 5 microns thickness were cut from each block. DNA from FFPE sections and cell line samples were extracted using the DNeasy blood and tissue kit (Qiagen, Hilden, Germany) as per the manufacturer’s instructions. DNA quantification was performed using the Qubit dsDNA HS Assay kit for the Qubit 2.0 Fluorometer (Life Technologies, Carlsbad, CA). The Qubit readings were used as a guideline for dilution of the DNA samples.

### FTH1 Taqman assay

FFPE derived DNA was checked for quality and concentration using an 180 bp *FTH1* TaqMan assay (Life Technologies). PCR was performed using the LightCycler 480 (Roche Diagnostics, Penzberg, Germany). The reaction mixture included 1X TaqMan Gene Expression Master Mix (Life Technologies), 1X *FTH1* TaqMan Assay labeled with FAM (Life Technologies, # 4331182), 1 μL of DNA and PCR grade water in a total volume of 11 μL. PCR conditions included an activation step of 10 min at 95°C followed by 40 cycles of 95°C for 15 seconds and annealing for 60 sec at 60°C. Based on a standard curve generated from genomic DNA of known concentrations of 50, 25, 12.5, 6.25, 3.13, 1.56 , 0.78 and 0.39 ng/μL and copy numbers per μL of 16667, 8333, 4167, 2083, 1042, 521, 260, 130 respectively, the copy numbers per μL for each sample were calculated according to the LightCycler 480 software manufacturer’s instructions.

### TruSeq amplicon cancer panel

The TruSeq Amplicon - Cancer Panel (TSACP) (Illumina, San Diego, CA) comprises 212 amplicons from 48 genes that are simultaneously amplified in a single-tube reaction. We used 5 μL of each DNA sample (50 ng/μl) for the experiment according to the manufacturer’s instructions. We used the MiSeq system (Illumina) for paired end sequencing with a v2 150-bp kit.

### Uracil-DNA glycosylase treatment

For samples treated with uracil-DNA glycosylase (UDG), FFPE DNA was dispensed and reduced to a final volume of 2 μL by vacuum centrifugation. The DNA was incubated with 6.25 U (1 U/20 ng of DNA) UDG (New England Biolabs, Ipswich, MA) in a final volume of 20 μL containing 1 U UDG buffer. After an initial incubation at 37°C for 2 h, the UDG enzyme was inactivated at 95°C for 10 min. UDG-treated FFPE DNAs were stored at 4°C before use in the TSACP reactions. Before use in sequencing, the volume of the reaction was reduced to a final volume of 5 μL by vacuum centrifugation.

### NRAS mutation testing

To confirm the *NRAS* negative result in sample Ca97, a second MPS method using deep sequencing was also used to assess exon 3 of the *NRAS* gene. Briefly, PCR was performed on a Fluidigm Access Array according to standard protocols [Access Array™ System for Illumina Sequencing Platform User Guide (PN 100–3770)] with the same FFPE sample (50 ng/μl) loaded into one well of the Access Array. The primers were 5’-acactgacgacatggttctacaGAGACAGGATCAGGTCAGCG-3’ and 5’-tacggtagcagagacttggtctGATGTGGCTCGCCAATTAAC-3’, giving an amplicon size of 253 bp (CS1 and CS2 tags are shown in lower case). After barcoding, the sample was run on an Illumina Miseq according to the manufacturer’s instructions (v2 150-bp kit).

### Bioinformatics

CASAVA v1.8.2 was used to perform sample de-multiplexing and to convert BCL files generated from the MiSeq instrument into Fastq files containing short-read data. Using the primer sequences that are present in the data, short reads were first assigned to their respective amplicon. Global alignment based on the Needleman–Wunsch algorithm was then performed between the reads and the amplicon reference sequences to identify sequence variations. Likely true variants were identified by *a*) VarScan2 and *b*) a variant frequency of >10% and were not included in the analysis of sequencing artefacts. Variants with a frequency < 1% were assumed to be sequencing errors. The remaining base changes (i.e. all except true biological variants and sequencing errors) were counted and categorised into respective nucleotide groups using custom Python scripts. In addition to the 1-10% allele frequency range, the same pipeline was also used to determine artefacts in the 10-25% allele frequency range.

### Statistical analysis

Spearman tests were used to examine associations between continuous variables. The Kruskal–Wallis one-way analysis of variance followed by the Dunn’s *post hoc* test was used to test for associations between continuous variables (C>T/G>A changes, *FTH1* results) versus anatomical pathology laboratory and tumour type. All analyses were performed using GraphPad Prism software version 6.01.

## Results

### Cell lines and FFPE samples

This study used the data from the first 488 patients recruited from the pilot phase of the Cancer 2015 study (Table 1). The FFPE samples analysed in this study were relatively recent (median: 77 days, mean: 93.4 ± 77 days) with a range of 4 to 851 days post fixation (Additional file 1: Table S1). HL-60 cell line DNA was used in each sequencing run and allowed run-to-run differences to be compared in terms of coverage and sequencing artefacts.

**Table 1:** Summary of formalin-fixed samples

**Tumour type**	**Number of cases**	**Percentage**
Breast	81	16.6%
Head and neck	80	16.4%
Prostate	79	16.2%
Colorectal	52	10.7%
Lung	47	9.6%
Other*	42	8.6%
Cervical	25	5.1%
Bone and soft tissue	22	4.5%
Oesophagogastric	15	3.1%
Renal	14	2.9%
Central nervous system	12	2.5%
Melanoma	11	2.3%
Cancer of unknown primary	8	1.6%

*Represents other cancer types with smaller numbers including pancreatic, ovarian, thyroid, testicular, bladder, hepatic, endometrial, biliary and anal cancers.

### Assessment of fragmentation in FFPE samples

**Figure 1:**
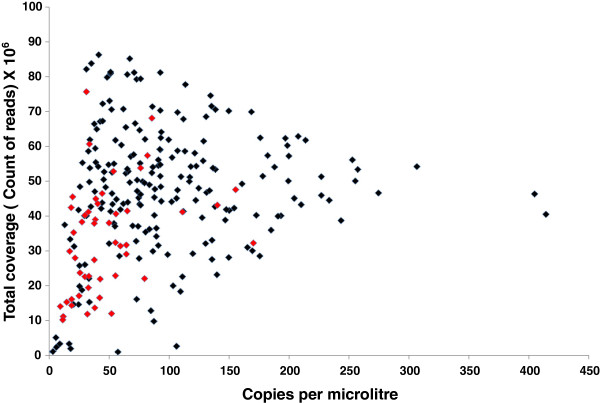
**Association of fragmentation of DNA in FFPE samples with low sequencing coverage.** Coverage of each sample (number of reads) versus the copies of the *FTH1* gene as assessed by a Taqman PCR assay (copies per microlitre). n = 253. There was a positive correlation between the *FTH1* result and coverage (Spearman correlation, r = −0.29, p < 0.0001). The 50 samples with the highest C>T/G>A levels in the 1-10% allele frequency range are shown in red.

The quality and quantity of DNA extracted from tumour biopsies can vary substantially between samples. DNA fragmentation is a major form of DNA damage that can be assessed through a number of quality control assays that measure if a minimal length of template can be amplified. Part of the pilot phase of the Cancer 2015 study was to assess the feasibility of using FFPE DNA for multiple parallel sequencing using the TSACP assay. The average length of amplicons in the assay is ~175 bp (range 152–189 bp). A subset of samples (n = 253) was assessed for amplifiable copy numbers using the commercially available Taqman assay that measures the copies of the *FTH1* sequence per microlitre of a sample based on the amplification of an 180 bp product. As expected, FFPE samples showed a large spectrum in the estimated number of copies of the *FTH1* sequence (Figure 1 and Additional file 1: Table S1). The cell line DNA samples showed good copy numbers on the *FTH1* assay (mean copies per microlitre were 152 and 160 for the HL-60 and H-1975 cell lines respectively at 50 ng/μl).

Overall total coverage of a sample after sequencing would be expected to be inversely associated with the amount of fragmentation whereby samples with high level of DNA fragmentation would have less amplification and therefore associated with lower coverage. By comparing both the *FTH1* and coverage results for all FFPE samples, there was a weak but significant association between these two variables (r = 0.29, p < 0.0001, Spearman Rank correlation). Given a minimum of 200,000 total reads to achieve a mean of ~1000X across all amplicons in a sample, 55/488 samples would not achieved this level of coverage.

There were two outlier samples that displayed high amounts of amplifiable template as measured by the *FTH1* assay but resulted in low coverage (samples Ca23 and Ca156). These samples however could have low coverage because of experimental error during the processing of the sample resulting in low coverage. However, for the overwhelming majority of other samples, there was a noticeable trend with the degree of fragmentation as measured by the *FTH1* assay and the resulting coverage after sequencing. Importantly, this indicates that significant fragmentation is present even in relatively recently fixed biopsies and is an important factor leading to lower amounts of successful sequencing data.

### Assessment of sequencing artefacts in FFPE samples

While fragmentation is a well known form of DNA damage that is present in a large number of FFPE samples, sequencing artefacts as a result of PCR amplification have not been extensively studied, especially in relatively recent samples as would be used diagnostically.

Hence, our next analysis was to determine if sequence artefacts were also present in our cohort of recently fixed samples. We previously published an informatic pipeline to assess the level of artefacts in archival lung squamous cell carcinoma samples [12]. This pipeline identifies counts of nucleotide changes within the 1-10% frequency range and removes counts from likely true mutations that are identified through the Varscan2 caller. Using this pipeline, we measured the degree of artefacts between the 1-10% range for all 488 FFPE samples and cell line samples.

**Figure 2:**
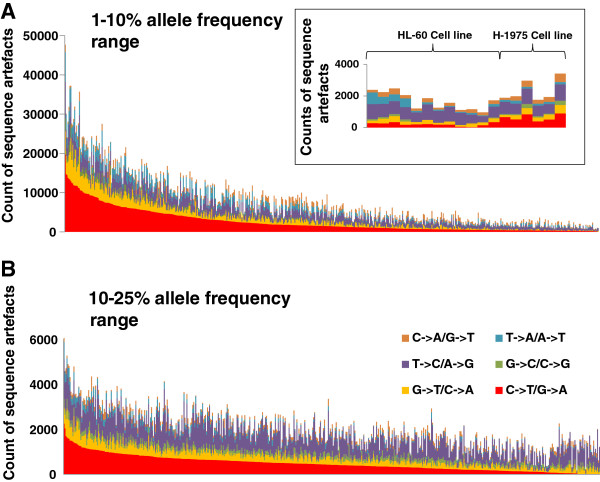
**Significant levels of C>T/G>A sequencing artefacts in FFPE samples. (A)** Assessment of sequence artefacts in cell line DNA and FFPE samples. The prevalence of each type of nucleotide change in the 1-10% allele frequency range was computed. Likely true variants identified through the Varscan2 variant caller were operationally removed to enrich for sequencing artefact changes. The graph shows all FFPE samples sorted according to the counts of C>T/G>A changes. Zoomed view: HL-60 and H1975 cell lines were used as good quality DNA controls. **(B)** The prevalence of each type of nucleotide change in the 10-25% allele frequency range. The graph shows all FFPE samples sorted according to the counts of C>T/G>A changes.

The results of the cell line samples are shown in Figure 2A (zoomed view) and illustrate no dramatic C>T/G>A changes observed at this 1-10% allele frequency range. There was however a consistently higher rate of C>T/G>A changes in the H1975 cell line compared to the HL-60 cell line suggesting low level heterogeneity that was not removed by the pipeline. Coverage for the cell line samples was sufficient for all samples and quite similar between runs [mean 52 million reads ± 13 million reads (standard deviation), n = 12]. Counts for sequencing artefacts were also very low and consistent. This indicates no major run-to-run bias.

In contrast, the FFPE samples showed a large range of C>T/G>A changes in a large proportion of samples (Figure 2). The increase in C>T/G>A changes was not discrete and not confined to only a subset of samples but appeared to be continuous suggesting that this type of artefact is ubiquitous in nature and will occur to some extent in every FFPE sample. The relative proportion of C>T/G>A changes was highly significant compared to other nucleotide changes (p < 0.0001, Kruskal–Wallis one-way analysis of variance) and formed 32% of all nucleotide changes overall, a proportion that was double what was expected due to chance.

By contrast, the same analysis, applied to look at possible artefacts between the 10-25% allele frequency range, indicated that C>T/G>A changes appear at much lower levels compared to the 1-10% allele frequency range (the scale on the y axis of Figure 2B is much smaller than that of 2A) and most likely represent real variants that were not be removed using our algorithm. Importantly, there was no observable bias towards C>T/G>A changes (Figure 2B).

Assuming that cell line DNA represents good quality DNA that would display little or no artefacts, the median C>T/G>A change count in the 1-10% allele frequency range was 272 contrasting to a much more higher median C>T/G>A count in FFPE samples of 1515. These findings not only confirm our original findings that C>T/G>A are the most predominant type of sequencing artefact but also indicate that they occur in all FFPE samples to some degree.

### Correlation of sequence artefacts with coverage

An interesting observation in samples with high C>T/G>A changes in the 1-10% range was a parallel increase in the number of other nucleotide changes. This could be due to the stochastic nature of low template samples which increase the probability for not only C>T/G>A artefacts but also other nucleotide artefacts to appear.

**Figure 3:**
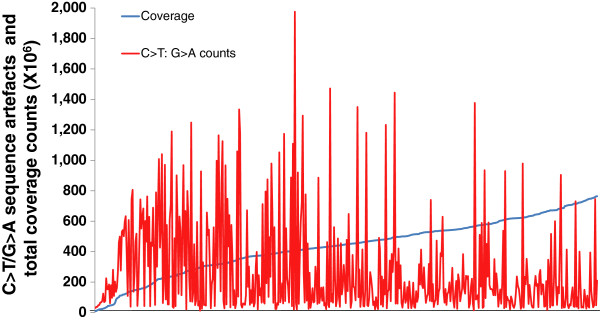
**Low coverage samples have higher rates of C>T/G>A sequencing artefacts.** For all FFPE samples (x-axis), values for coverage (blue) and the counts of C>T/G>A sequencing artefacts (red) are plotted on the same y-axis. There was an inverse correlation between coverage and C>T/G>A sequence artefacts (Spearman correlation, r = −0.24, p < 0.0001).

To confirm this phenomenon, the coverage and counts for C>T/G>A changes were graphed for each FFPE sample (Figure 3). As expected, there was a significant association between low coverage and high amounts of C>T/G>A changes in the 1-10% allele frequency range (r = −0.24, p < 0.0001, Spearman Rank correlation). This is also in line with the fragmentation measurements with the 50 samples with the highest C>T/G>A levels strongly trending towards having lower estimated template numbers in the *FTH1* assay (Figure 1). This indicates that despite the same amount of DNA used for each sample that the reduction of available templates caused by fragmentation can lead to a higher probability for sequence artefacts to be observed after PCR amplification.

### Correlation of anatomical laboratory with sequence artefacts and coverage

While DNA was extracted at the same location with the same protocol, the tissue blocks were formalin fixed and paraffin embedded in different anatomical pathology laboratories across Victoria, Australia. Since different anatomical laboratories might have different practices regarding the fixation of tissues, we examined DNA damage in relation to the originating anatomical laboratory. There was a significant difference for both fragmentation and sequence artefacts between anatomical pathology (AP) laboratories (p < 0.0001, Kruskal-Wallis test). Dunn’s Multiple Comparison test indicated significant difference between AP7 versus AP22 and AP26 for fragmentation analysis, and AP7 versus AP22 for sequencing artefacts (Additional file 2: Figure S 1). Despite standardization, these results suggest that fixation processes may vary across different labs. The age of the blocks or the tumour type was not significantly correlated with the degree of fragmentation or sequence artefacts.

### Case studies: samples with potential false positive clinical mutation calls

**Figure 4:**
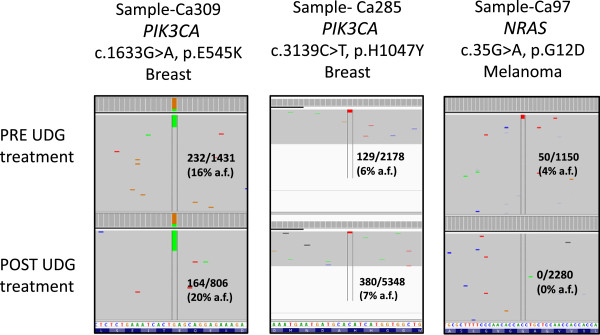
**Uracil-DNA glycosylase treatment of FFPE DNA samples distinguishes true and false positive clinical relevant mutations.** Integrative Genomic Viewer (IGV) screenshots of two breast cancers and one melanoma sample pre- and post- uracil-DNA glycosylase (UDG) treatment samples. The two breast cancer samples have confirmed *PIK3CA* mutations (E545K for Ca309 and H1047Y for Ca285) as these mutations were still detected after UDG treatment. The *NRAS* G12D mutation identified in the pre-UDG sample (Ca97) was a false positive as it was not present after UDG treatment. The variant reads over the total reads and overall allele frequency (a.f.) are shown for each case.

To determine if samples with high artefacts contain mutations that mimic actionable mutations, we selectively examined the variant calls from samples with high artefact rates. From this, we found three samples that had actionable mutations with low frequencies and with enough available DNA for further testing. We treated these samples with uracil-DNA glycosylase (UDG), sequenced them using the TSACP and then examined if the mutation was still present. As shown in Figure 4, *PIK3CA* mutations in the two breast cancer cases (Ca309 and Ca285) were confirmed to be real mutations as the identical mutation of similar frequency was identified in each of these cases after UDG treatment. *PIK3CA* mutations are positive prognostic factors in breast cancer [21,22], supporting the clinical utility of MPS for the detection of clinically actionable mutations. It also illustrates that this platform can detect real mutations even at low frequencies.

In the case of a melanoma with an apparent *NRAS* c.35 G>A, p.G12D mutation (Ca97), resequencing (after UDG treatment) using the TSACP platform did not confirm the same mutation. To further validate this result, the sample was tested by an orthogonal MPS method (Fluidigm Access Array microfluidic chip system) that covers the *NRAS* exon 2 region at extremely high coverage (Additional file 2: Figure S2). This method also could not detect the mutation suggesting that the original call for the mutation was a false positive.

## Discussion

While the implementation of MPS into a clinical setting is currently in progress in numerous laboratories world-wide, there are still a number of challenges using DNA from FFPE cancer specimens for this application. The prevalence of DNA damage associated with formalin fixation in clinical material has not been extensively studied, particular in terms of cancer genomics. Most genomic studies dealing with FFPE DNA are relatively small in numbers with the archival status of specimens in most cases remaining unknown [4,16]. In contrast, this study had a relatively narrow window in which samples were formalin-fixed and had a standardised and centralised point of DNA extraction and sequencing. FFPE samples in this study therefore accurately represent those that would enter a molecular diagnostic clinical workflow.

To our knowledge, this study represents the largest assessment of formalin induced fragmentation and sequencing artefacts using clinical FFPE samples. From our set of analyses, we have confirmed that both fragmentation and sequencing artefacts are common forms of DNA damage from formalin-fixed material even if blocks are relatively new. As a result, this work illustrates these forms of DNA damage can have major consequences for downstream interpretation as not all actionable mutations could be validated.

There are a limited number of publications highlighting the ability of amplicon-based MPS to identify clinically relevant mutations or canonical mutations from DNA obtained from various tumour types [23-26]. However, these studies looked at a relatively small number of cases, usually reported only canonical mutations, and where validation of variants was performed, demonstrated variation in the results obtained. For example, in the study investigating the sensitivity of immunohistochemistry (IHC) versus Sanger sequencing to *BRAF* V600E mutations in FFPE derived DNA from papillary thyroid carcinoma specimens, Bullock and colleagues utilised the TSACP as an alternative sequencing platform [23]. The TSACP was able to identify the *BRAF* V600E mutation in three out of 11 IHC positive cases with variant frequencies of 10-32%, which Sanger sequencing failed to detect. When DNA was then macro-dissected from the same tumour blocks, both sequencing and the TSACP analyses could now both detect the V600E mutation in seven cases (7/11). This confirms that the platform is comparatively more sensitive than the “gold standard” of Sanger sequencing for low frequency mutations.

In this study we explored two common form of DNA damage caused by formalin, i.e. fragmentation and sequencing artefacts. Assessment of fragmentation was performed using a copy number assessment PCR prior to MPS. The weak association between total coverage counts and the *FTH1* results could be explained by the limitations of the *FTH1* assay. While the assay can clearly discriminate between samples based on the degree of fragmentation, the assay is designed only around a product of 180 bp and for only one locus. Consequently, the *FTH1* assay cannot accurately assess the entire range of product sizes generated by the TSACP protocol. Testing multiple loci across various fragment lengths is therefore recommended for FFPE samples in MPS testing to predict poor quality amplifiable DNA [27].

Interestingly, the degree of C>T/G>A artefacts forms a continuum suggesting that the source of DNA damage causing these changes occurs to some degree in every sample. Procedurally, although the practice of fixation of tumour specimens is standardised, the observation that samples processed from one anatomical pathology laboratory (AP7) had a significantly higher rate of sequencing artefacts and fragmentation indicates that fixation processes may not actually be uniform between different laboratories.

Assessment of other variables potentially involved in DNA damage such as the duration of transport from patient to the anatomical pathology laboratory, and size of tumour specimens could not be feasibly assessed in this study. In addition, the degree and spectrum of DNA damage from a formalin-fixed sample depends on environmental factors such as exposure to heat, light and the concentration and age of formalin used for fixation. The age of blocks examined is another consideration as long-term storage in suboptimal environments can cause significant DNA damage. Further dissection of these factors in a more controlled setting will provide significant benefits to the preservation of DNA for subsequent testing.

Another consideration is the type of polymerase used in this study. While the type of polymerase used in the TSACP was not disclosed due to commercial reasons it is probably not proofreading since proofreading enzymes are known to stall at uracil lesions and in effect, replicate the effect of UDG treatment. Further examination of polymerases which increase correct sequence amplification but still maintain sufficient amplification for sequencing are required.

There is a possibility that some changes within the 1-10% allele frequency do represent low-level heterogeneity and were not detected by the Varscan2 variant caller and removed from our analysis of sequencing artefacts. Fresh frozen samples counterparts would be suitable for this assessment but were not available for this study to confirm this.

In this study, we confirmed the detection of two low frequency actionable mutations in the *PIK3CA* gene, in breast carcinoma cases. These mutations remained after the sequencing was repeated (and after UDG treatment). Given the clinical context of these mutations in breast cancer, these mutations are of potential clinical benefit to the patient that may have implications for *PIK3CA* inhibitors that target the PI3K/AKT/mTOR pathway [21,22]. It also illustrates that this platform can detect real mutations even at low frequencies.

In contrast, an activating *NRAS* G12D mutation discovered originally in our first screen but not confirmed in the same DNA specimen by subsequent UDG treatment or MPS has major implications for the patient. Activating mutations in *NRAS* have been reported in approximately 20% of all melanomas [28] and are potentially sensitive to therapeutics that target downstream signaling through mitogen-activated protein kinase kinase and phosphatidylinositol 3-OH kinase or AKT [29,30]. This is a clear demonstration of the dangers that can arise from MPS data without proper validation.

Complete validation of all variants was not possible in this study because of the extremely large number of variants detected and the lack of remaining DNA available for all samples. Undoubtedly a large number of these variants will be false positives, especially in the 1-10% allele frequency range. While validation of every variant is a laborious task costing time, material and expense, we recommend that validation of actionable mutations that will directly affect patient management be performed in this kind of testing. We also propose that quality control measures be adopted prior to sequencing including the implementation of more efficient and accurate quality control assays that evaluate DNA concentration, fragmentation and the presence of uracil lesions.

While we have shown treatment with UDG is effective in reducing artefactual variants, there are a number of other strategies to minimise artefacts occurring, particularly those using non-capture based-technologies [31]. This includes performing duplicate reactions for the same sample or through internal validation by having overlapping amplicons covering the same loci (as is the case in the newer TruSight™ version of the TSACP panel that was released after this study was concluded).

## Conclusions

**Figure 5:**
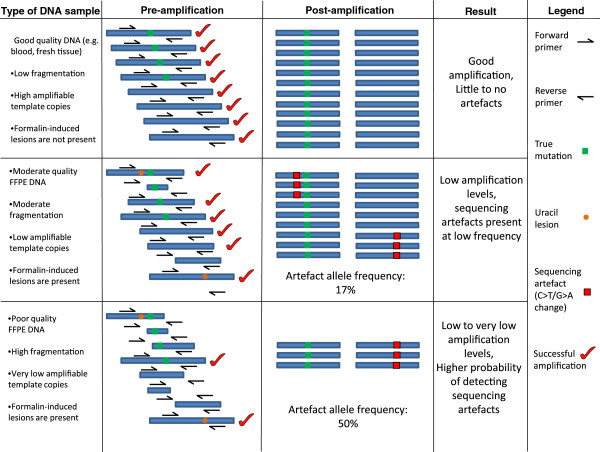
**Low template copies are associated with higher probability of sequencing artefacts post-PCR amplification.** In good quality DNA from sources such as blood and fresh frozen tissue, fragmentation and uracil lesions are present at very low levels. In this circumstance, high amounts of amplifiable template increase the likelihood of accurately identifying mutations due to high sequencing coverage with little or no stochastic enrichment of sequencing artefacts. In FFPE DNA with moderate fragmentation, the number of amplifiable templates is reduced, with some formalin-induced uracil lesions being present in template DNA. Subsequently PCR amplification results in lower coverage due to less amplifiable template numbers. Uracil lesions are also amplified, and due to the lower copy numbers, can appear as non-reproducible sequencing artefacts (C>T/G>A changes). These artefacts will be low in frequency. In the case of FFPE with high amounts of fragmentation, the numbers of amplifiable template are severely limited. An artefact in one of these templates can then appear as a moderate to high frequency sequencing variant. These can subsequently be interpreted as real mutations.

DNA damage caused by formalin fixation appears to be very common even in relatively recent samples. This study demonstrates that higher amounts of fragmentation will increase the probability of higher rates of sequencing artefacts. This is summarised in Figure 5. The findings from this study not only have implications for MPS-based platforms but also conventional PCR-based methodologies which commonly use FFPE DNA as an input such as HRM and Sanger sequencing. Samples with a high degree of DNA damage must be treated with caution as potential false positives that can arise from formalin damage may have major consequences for downstream clinical decisions. As MPS becomes increasingly incorporated into clinical diagnostic workflows, it is important to assess DNA damage caused by formalin fixation, as this will greatly optimise diagnostic workflows, increase accuracy of results and lead to better outcomes for patients.

## Competing interests

GM has received commercial research funding from Pfizer, Novartis, and Millennium. AD has received commercial research funding from Pfizer.

## Authors’ contributions

SW, HD, JP and AD conceived the study. SW, AT and RV performed the experiments. SW, JL, JP, JE, KD and AD analysed the data. JP, SF, AF and AB provided pathological interpretation and material support. GM and DT provided the clinical interpretation. SW and AD wrote the manuscript. All authors contributed to revision of the manuscript and approved the final version.

## Pre-publication history

The pre-publication history for this paper can be accessed here:

http://www.biomedcentral.com/1755-8794/7/23/prepub

## Supplementary Material

Additional file 1: Table S1 Coverage, *FTH1* results, counts of C>T/G>A changes in the 1-10% allele frequency range, anatomical pathology lab, tumour type and age of block. Click here for file

Additional file 2: Figure S1 Fragmentation and sequencing artefacts for each anatomical pathology laboratory. **Figure S2.** Negative *NRAS* exon 2 mutation result for a melanoma case. Click here for file
