# Supplementary material for: Sequence artefacts in a prospective series of formalin-fixed tumours tested for mutations in hotspot regions by massively parallel sequencing
Source: BMC Med Genomics. 2014 May 13;7:23. doi: 10.1186/1755-8794-7-23 (PMC4032349; doi:10.1186/1755-8794-7-23)
Supplement: Additional file 2: Figure S1 — Fragmentation and sequencing artefacts for each anatomical pathology laboratory. Figure S2. Negative NRAS exon 2 mutation result for a melanoma case. [file 1755-8794-7-23-S2.ppt]

## Slide 1
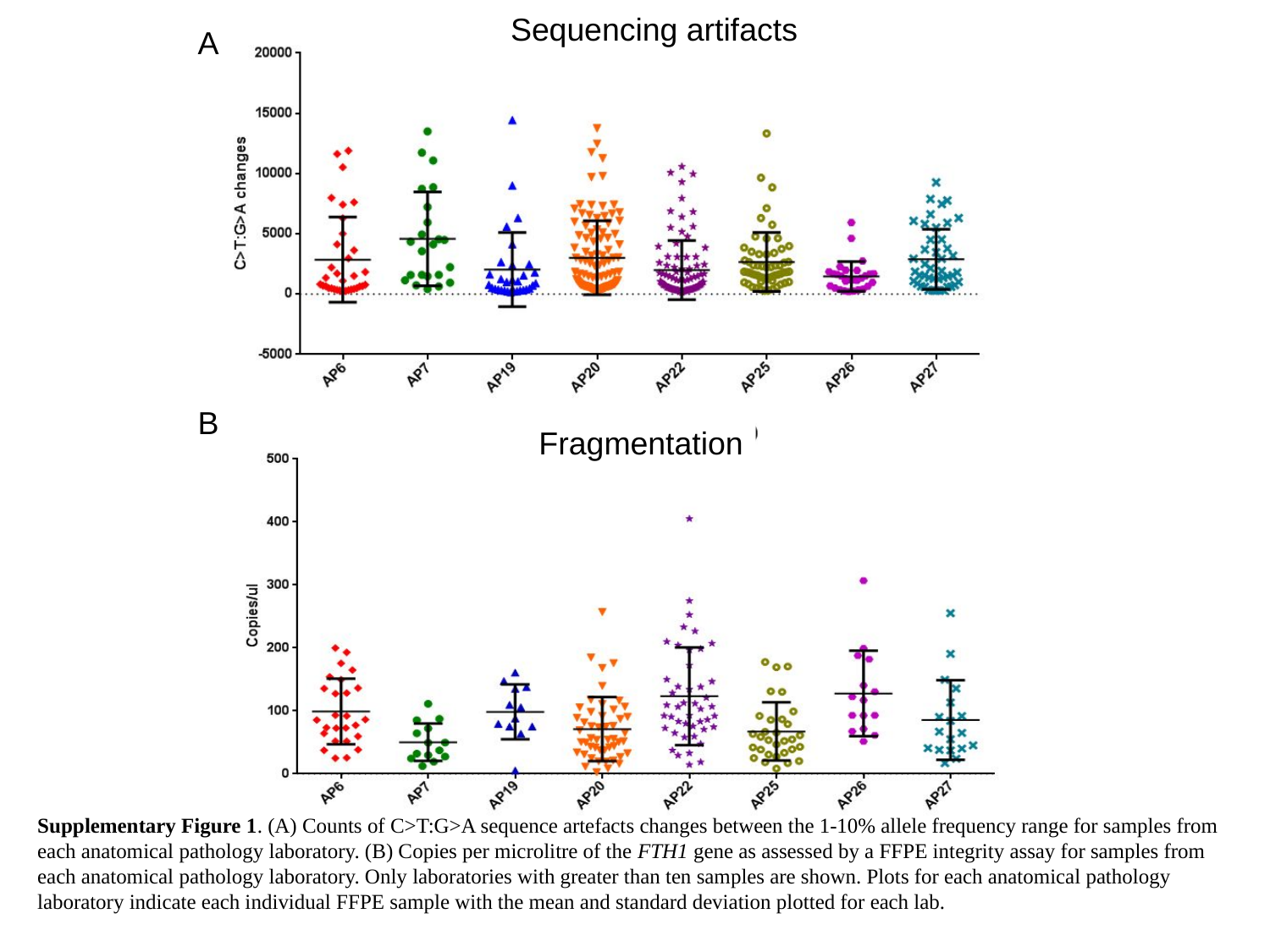

Sequencing artifacts
A
B
Fragmentation
Supplementary Figure 1. (A) Counts of C>T:G>A sequence artefacts changes between the 1-10% allele frequency range for samples from each anatomical pathology laboratory. (B) Copies per microlitre of the FTH1 gene as assessed by a FFPE integrity assay for samples from each anatomical pathology laboratory. Only laboratories with greater than ten samples are shown. Plots for each anatomical pathology laboratory indicate each individual FFPE sample with the mean and standard deviation plotted for each lab.

## Slide 2
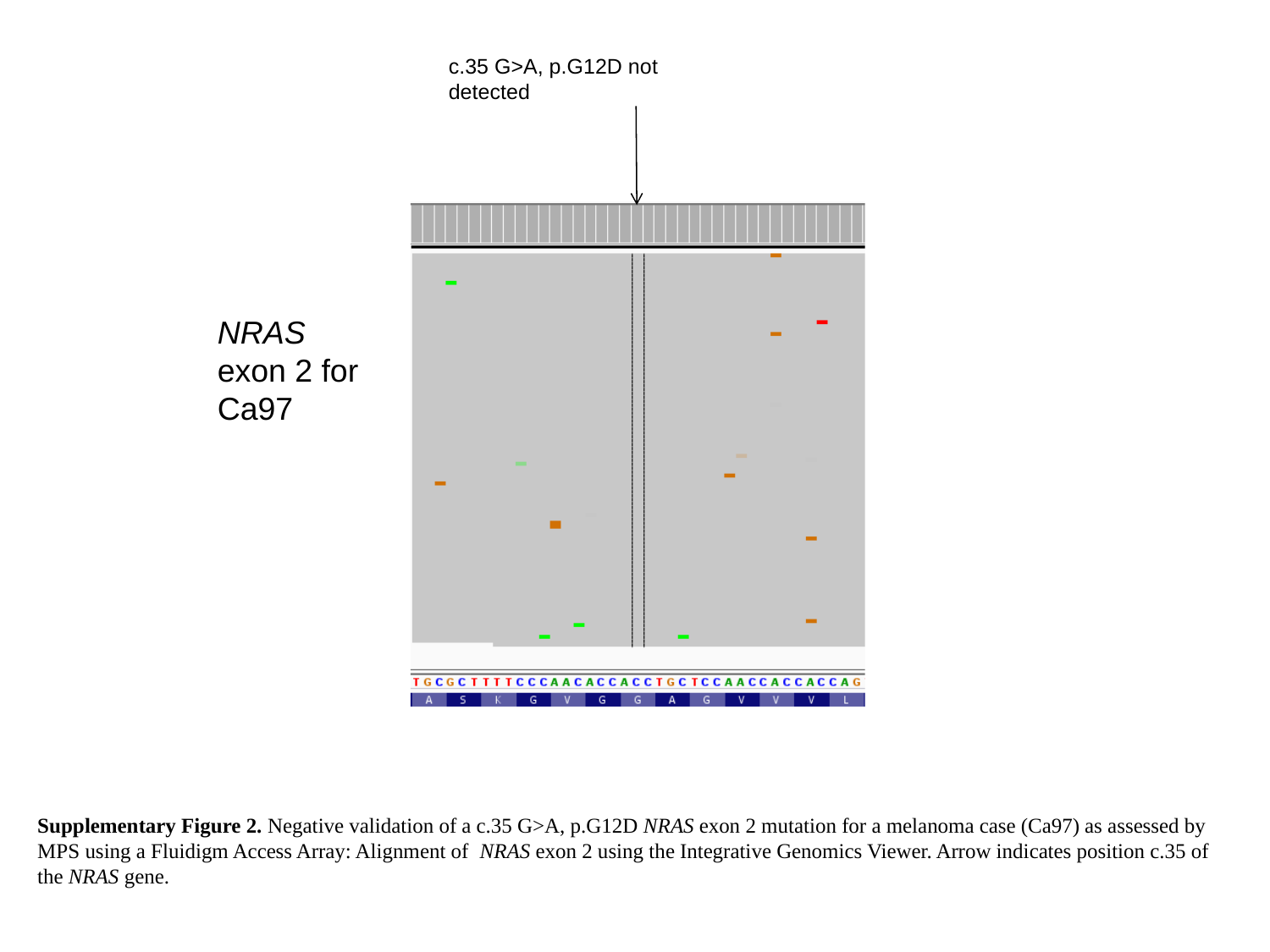

c.35 G>A, p.G12D not detected
NRAS exon 2 for Ca97
Supplementary Figure 2. Negative validation of a c.35 G>A, p.G12D NRAS exon 2 mutation for a melanoma case (Ca97) as assessed by MPS using a Fluidigm Access Array: Alignment of NRAS exon 2 using the Integrative Genomics Viewer. Arrow indicates position c.35 of the NRAS gene.
